# Verb vocabularies are shaped by complex meanings from the onset of development

**Published:** 2023

**Authors:** Justin B. Kueser, Arielle Borovsky

**Affiliations:** Department of Speech, Language, and Hearing Sciences, Purdue University, West Lafayette, IN 47907 USA

**Keywords:** vocabulary development, semantic features, semantic networks, verbs, nouns, complexity

## Abstract

Verbs and nouns vary in many ways – including in how they are used in language and in the timing of their early learning. We compare the distribution of semantic features that comprise early-acquired verb and noun meanings. Given overall semantic and syntactic differences between nouns and verbs, we hypothesized that the preference for directly perceptible features observed for nouns would be attenuated for verbs. Building on prior work using semantic features and semantic networks in nouns, we find that compared to early-learned nouns (N = 359), early-learned verbs (N = 103) have meanings disproportionately built from complex information inaccessible to the senses. Further, children’s early verb vocabularies (N = 3,804) show semantic relationships strongly shaped by this complex information from the beginning of vocabulary development. Complexity is observed in early verb meanings and is reflected in the vocabularies of children even at the outset of verb learning.

## Introduction

Verbs demonstrate significant semantic differences from nouns that may influence early vocabulary development. For example, compared to nouns, verbs are associated with less concrete and imageable entities (Gentner, 1982; Ma, Golinkoff, Hirsh-Pasek, Mcdonough, & Tardif, 2009; McDonough, Song, Hirsh-Pasek, Golinkoff, & Lannon, 2011; Smolík, 2019) and may describe transient or complex events involving many interacting participants (Talmy, 1985). In a variety of languages, including English, verbs tend to be learned later than nouns (Frank, Braginsky, Yurovsky, & Marchman, 2021).

Evidence suggests that simple and accessible information is preferred in early vocabularies. Early-learned nouns, for instance, tend to have high concreteness and imageability (e.g., Hansen, 2017). Early verbs also demonstrate similar patterns favoring easily accessible perceptual information. For example, perceptual similarity of participants across events helps toddlers extend novel verbs to dissimilar participants (Childers et al., 2016; Haryu, Imai, & Okada, 2011).

Nevertheless, verb meanings may comprise different kinds of information than noun meanings. Given that verbs are less imageable and concrete than nouns, verb meaning may reflect less directly accessible semantic information compared to nouns. As such, early verb learning requires learning about the wealth of verb-specific semantic detail involved in verbs’ thematic roles. For example, adults know that the meaning of the verb “frighten” includes knowledge about its patient – not only basic facts that the patient is likely affected (Dowty, 1991) – but also specific information that things that are frightened tend to be “scared”, “small”, “weak”, and “helpless” (McRae, Ferretti, & Amyote, 1997). While some parts of this information are directly perceptible (e.g., “small”), other parts implicate more advanced knowledge (e.g., “helpless”). The extent to which such socially determined, complex information is prioritized in early verb meanings is unknown but such information is likely pervasive.

In this study, we ask two questions about verb learning in young children. First, are the meanings of early-learned verbs composed of different kinds of semantic information than nouns? And second, how do different kinds of semantic information contribute to the semantic relationships among early-learned nouns and verbs?

Word meaning has been fruitfully decomposed into tractable subcomponents by developing and analyzing lists of semantic microfeatures that are produced by adults in response to concepts (McRae, Cree, Seidenberg, & McNorgan, 2005; Vinson & Vigliocco, 2008). For example, the semantic features of the word “tree” include features like <is tall> and <has leaves>. Semantic features account for patterns of word use and understanding in adults (e.g., McRae et al., 1997; Pexman, Hargreaves, Siakaluk, Bodner, & Pope, 2008) and children (e.g., Engelthaler & Hills, 2017; Hills, Maouene, Maouene, Sheya, & Smith, 2009; Peters & Borovsky, 2019; Stella, Beckage, Brede, & De Domenico, 2018).

Semantic features can be categorized according to the type of information they encode (Cree & McRae, 2003). Perceptual features encode sensorimotor information about meaning (e.g., <is red>, <is greasy>). Functional features are associated with information about interactions with objects (e.g., <used for transportation>, <is eaten>). Taxonomic features refer to hierarchical relationships among concepts (e.g., <a vehicle>, <a food>). Finally, encyclopedic features are those that do not fit into the other categories (e.g., <is fun>, <is poisonous>) and are often associated with social factors, affect/emotion, and decontextualized knowledge (Cree & McRae, 2003). As such, encyclopedic features often describe complex meanings that cut across the other feature types. In fact, these features have occasionally been excluded from semantic-feature-based analyses of early vocabulary development because they have been assumed to be inaccessible and too complex (e.g., Hills et al., 2009), though we follow other work that includes them (Peters & Borovsky, 2019).

Among these feature types, perceptual and taxonomic features have been repeatedly implicated as shaping children’s early noun vocabularies (Engelthaler & Hills, 2017; Peters & Borovsky, 2019). For example, nouns with more perceptual features tend to have earlier ages of acquisition (AOAs), even after controlling for word frequency and the number of other feature types (Peters & Borovsky, 2019). Perceptual features may be particularly important to early vocabulary development because high concreteness (e.g., Gilhooly & Logie, 1980), imageability (e.g., McDonough et al., 2011), perceptual accessibility (Della Rosa, Catricalà, Vigliocco, & Cappa, 2010), and perceptual salience (Pruden, Hirsh-Pasek, Golinkoff, & Hennon, 2006) facilitate word learning and perceptual features tend to encode such information.

Perceptual features may also play a similar role for verbs. In general, verbs are better learned and recognized by young children when they have participants that are more familiar (Kersten & Smith, 2002) and more physically similar to those encountered in prior events (Childers et al., 2016; Haryu et al., 2011). These findings suggest that verb semantics in early vocabulary acquisition may be driven by perceptual information. It may only be later in vocabulary acquisition that non-perceptual information may support verb semantics. In general, the perceptual feature advantage in noun learning has been argued to reflect a perceptual-to-conceptual shift in language processing (Quinn & Eimas, 1997, 2000). This idea suggests that early noun learning depends on perceptually accessible features that are only later supplemented by more complex features, a transition that may also occur with verbs.

Alternatively, the four feature types may differently impact noun and verb semantics. This difference may be driven by differences in the concepts and actions that nouns and verbs refer to in events. Verbs often serve a coordinating role in sentences describing events, describing how one participant affects or interacts with another. Such interactions among participants may be particularly salient for younger children, who tend to prioritize associative or functional links among objects (e.g., <is eaten>) in some semantic processing tasks (e.g., Smiley & Brown, 1979).

The potential importance of functional features for verbs is consistent with theories suggesting that children undergo a thematic-to-taxonomic shift in vocabulary development (Inhelder & Piaget, 1964; Smiley & Brown, 1979). Under this theory, early verb semantics may expose event associations between verbs and nouns through functional features. For example, an early-learned verb like “eat” has clear functional relationships with a noun like “cookie” because both words tend to occur in similar event contexts like mealtimes. Such features could highlight likely relationships between objects and actions and therefore serve as important aspects of verb meaning. This idea is consistent with evidence that children’s early complex play behaviors are often associated with the typical functional uses of objects (e.g., Zelazo & Kearsley, 1980), suggesting that such functional information is important to children’s processing of events – and ultimately, their learning of verbs.

Encyclopedic features might also play a larger role in verb compared to noun learning. This idea is supported by findings that children need to learn the properties of verb meanings and associated event participants on a verb-by-verb basis (Alishahi & Stevenson, 2010; Meints, Plunkett, & Harris, 2008; Yuan, Fisher, Kandhadai, & Fernald, 2011). Such verb-specific features likely detail knowledge about common attributes of event participants like social roles, behaviors, or relationships, or emotional state and affect, that are not captured by other feature types. Work on school-age children suggests that the emotional valence of words influences their processing and learning, suggesting that such social and emotional information may also influence early verb learning (Ponari, Norbury, & Vigliocco, 2018, 2020).

Before we examine how feature type influences children’s early vocabularies, we first describe the overall vocabulary challenge faced by children by examining the relative feature composition of nouns and verbs in a large sample of early-learned words. We predicted that functional and encyclopedic features would make up relatively more of the feature composition of verbs compared to nouns and that perceptual and taxonomic features would be more abundant for nouns compared to verbs.

## Experiment 1

### Method

Semantic features for all of the nouns (N = 359) and verbs (N = 103) on the American English-language version of the MacArthur-Bates Communicative Development Inventory: Words and Sentences (MBCDI) were used (Borovsky, Peters, Cox, & McRae, Under review; Kueser, Horvath, & Borovsky, In prep.; McRae et al., 2005). The MBCDI is a checklist of early vocabulary items completed by caregivers for children aged 16 to 30 months of age. The semantic feature data sets were collected by asking adult participants to describe features of the words; the raw participant responses were standardized into semantic features shared across nouns and verbs. The resultant features were then categorized by type (Cree & McRae, 2003).

We counted the number of encyclopedic, functional, perceptual, and taxonomic features for each word. There were similar total raw numbers of features for nouns, *M* = 13.15, *SD* = 3.42, and verbs, *M* = 12.41, *SD* = 3.97, *t*(148.24) = 1.72, *p* = .088. To avoid any small differences in raw number biasing the results, we divided each count by the total number of features associated with each word, resulting in a set of proportions of the relative amount of encyclopedic, functional, perceptual, and taxonomic information for each noun and verb.

The relative proportion of each feature type across nouns and verbs was analyzed using ANOVA. The dependent variable was proportion and the independent variables were feature type and part of speech. Models and post-hoc tests were conducted using R version 4.1.1 (R Development Core Team, 2008) and the *emmeans* version 1.8.1–1 (Lenth, 2019)

### Results

**Figure 1:**
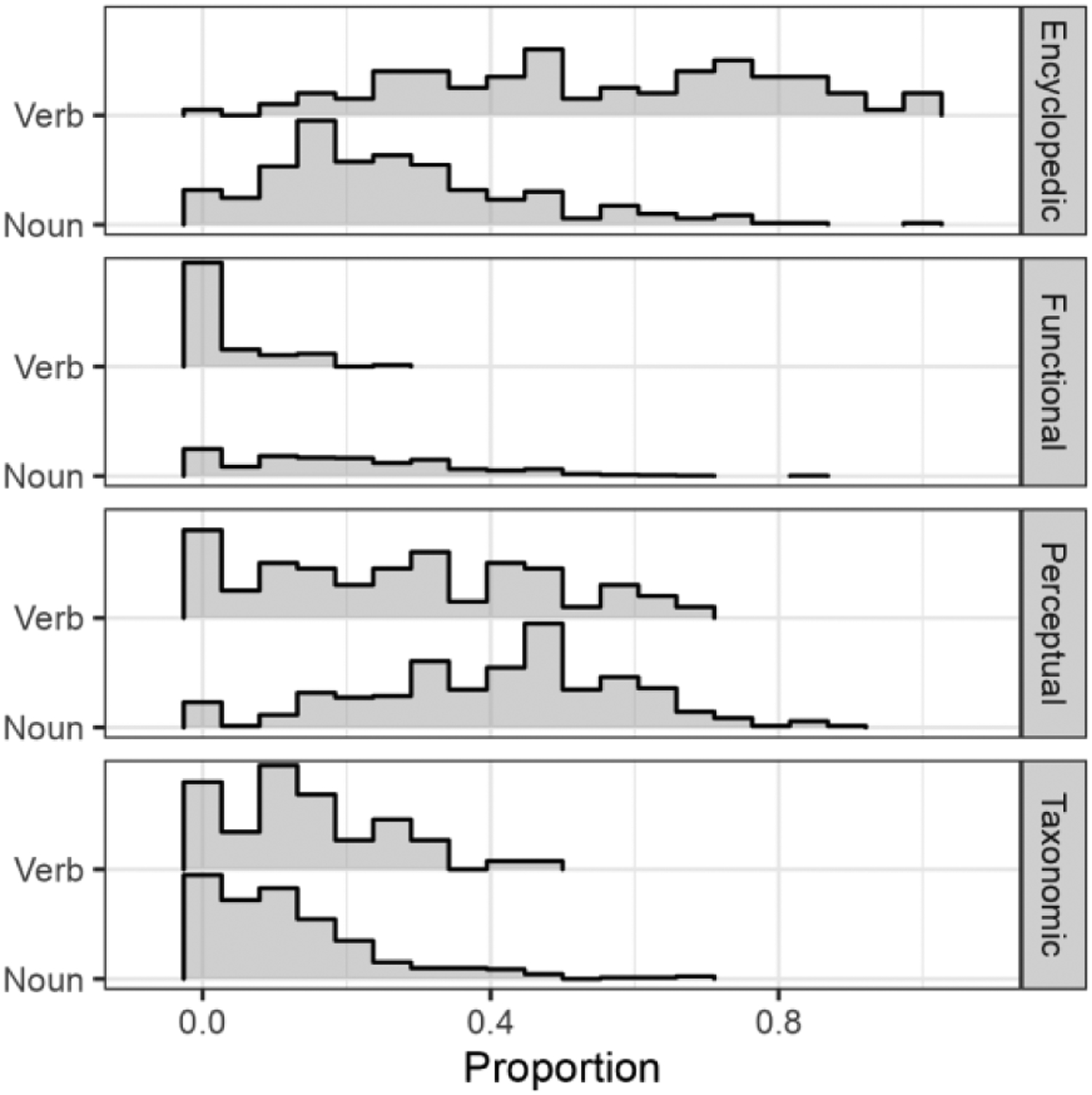
Histograms of proportion of features of each feature type relative to the count of all features for each early-learned noun and verb on the MBCDI. Data are binned in intervals of 0.05.

Figure 1 illustrates the relative proportion of features of each feature type across the MBCDI nouns and verbs. Several patterns emerged in the statistical analyses of these proportions, the results of which are presented in Table 1. There was a significant main effect of feature type *F*(3) = 261.5, *p* < .001. Across nouns and verbs, encyclopedic and perceptual features were most abundant. The average proportion of encyclopedic features relative to all features was 0.41, 95% CI: [0.39, 0.42]. The average proportion of perceptual features was 0.34, 95% CI: [0.33, 0.36]. The average proportion of taxonomic features was 0.13, 95% CI: [0.12, 0.15]. Last, the average proportion of functional features was 0.12, 95% CI: [0.10, 0.13].

The effect of feature type demonstrated a strong interaction with part of speech, *F*(3) = 125.7, *p* < .001. This interaction showed that encyclopedic features were more abundant for verbs than for nouns, EMM_N-V_ [estimated marginal mean] = −0.28, 95% CI: [−0.32, −0.25]. In contrast, functional features were more abundant for nouns than for verbs, EMM_N-V_ = 0.17, 95% CI: [0.13, 0.20], as were perceptual features, EMM_N-V_ = 0.13, 95% CI: [0.10, 0.17]. The proportion of taxonomic features was not significantly different for nouns compared to verbs, EMM_N-V_ = −0.02, 95% CI: [−0.05, 0.02]. Mean proportions by feature type for nouns and verbs are reported in Table 2.

## Experiment 2

Experiment 1 demonstrated that early-acquired nouns and verbs differ in their semantic feature composition. While both nouns and verbs tended to have proportionally more encyclopedic and perceptual features than functional and taxonomic features, verbs prioritized encyclopedic features and nouns prioritized perceptual and functional features. Overall, these patterns indicated that early-learned verbs’ meanings have more complex and less perceptually accessible information than early-learned nouns.

How might children use these noun-verb differences in semantic feature information during learning? On one hand, children may ignore complex semantic information like encyclopedic features and focus on more accessible perceptual information. Alternatively, given the preponderance of encyclopedic features in early-acquired verbs’ meanings, children might develop strategies to understand this more conceptually complex information. In order to expand their verb vocabularies, children may need to learn to consider aspects of meaning beyond directly perceptible features.

**Figure 2:**
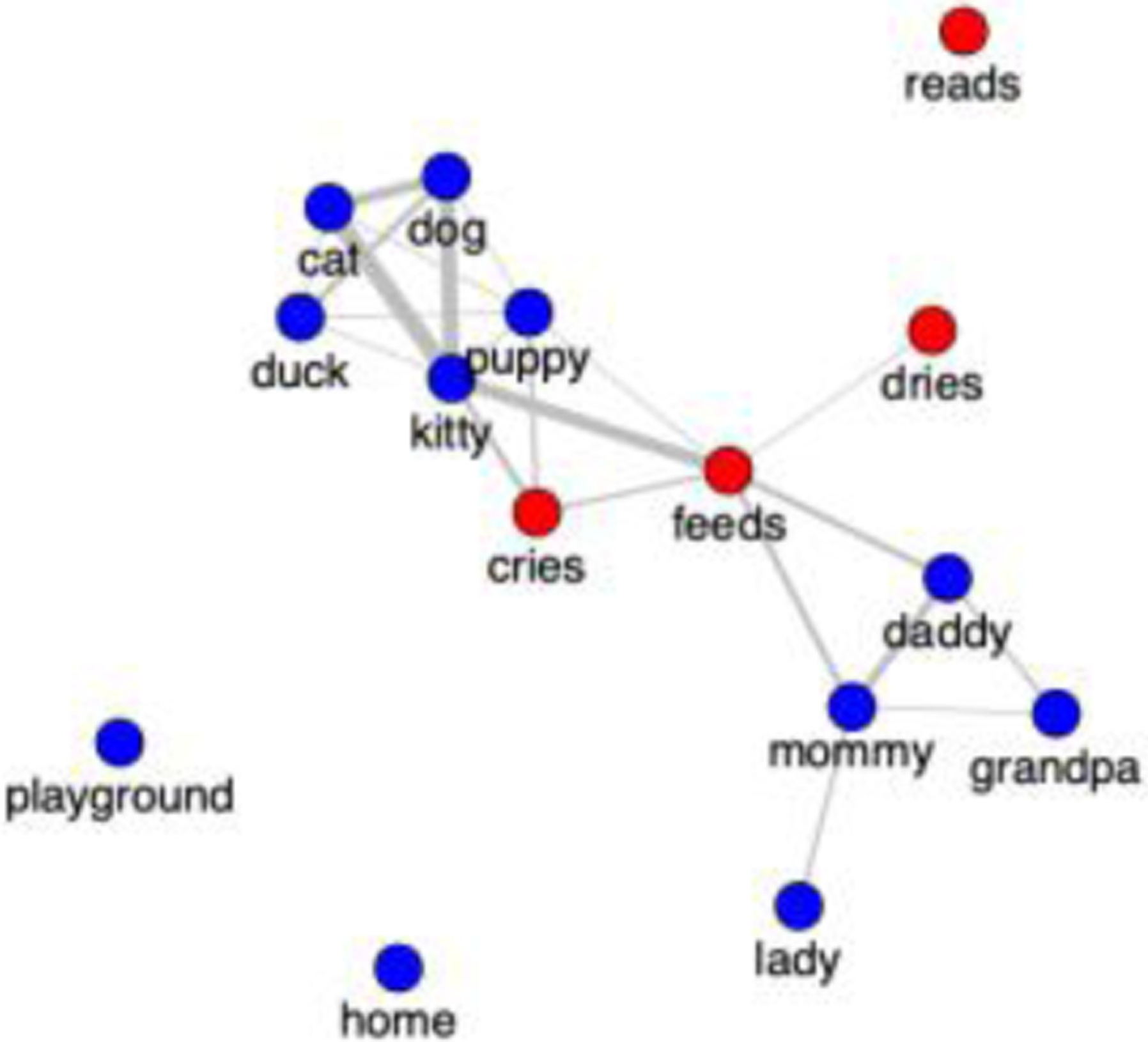
Example semantic network with nouns in blue and verbs in red. Shared semantic features connect words. Edge weight is the number of shared features.

Semantic network modelling can help to distinguish among these options. Semantic networks treat words as nodes in a network and connect words through shared semantic features. (Figure 2 shows an example semantic network.) Semantic network structure among words can be examined in this network by measuring the strength of semantic connections across the words in the network. For example, in Figure 2, “kitty” exhibits semantic structure characterized by strong semantic connections to its neighbors whereas “playground” has semantic structure characterized by having no semantic connections to any other word. Patterns of semantic structure in vocabulary networks predict words’ age of acquisition (Beckage & Colunga, 2019; Borovsky, Ellis, Evans, & Elman, 2016; Engelthaler & Hills, 2017; Fourtassi, Bian, & Frank, 2020; Hills, Maouene, Riordan, & Smith, 2010; Hills et al., 2009; Peters & Borovsky, 2019; Sailor, 2013; Stella et al., 2018; Steyvers & Tenenbaum, 2005) and other aspects of word processing (Borovsky, 2020, 2022; Peters, Kueser, & Borovsky, 2021). Examining patterns of semantic structure between nouns and verbs may help to identify which kinds of semantic features serve to connect these words to neighbors and which are prioritized in early learning.

Some evidence suggests that perceptual feature content may be a robust driver of semantic structure in early noun learning (Peters & Borovsky, 2019). In this study, the authors created semantic networks with the nouns on the MBCDI and their semantic relationships as defined by shared semantic features. Nouns that were directly connected to many other nouns through shared perceptual features tended to have earlier AOAs; the effect of other semantic feature types was less pronounced. Such a pattern may also occur for verbs.

However, given that Experiment 1 suggested that verb meaning more heavily relies on encyclopedic features, verb semantic structure may also reflect this tendency. Such a pattern might be seen in verbs’ semantic connections to other words being primarily composed of encyclopedic features compared to other features. Only one study has examined verb-specific network semantic structure (Kueser, Horvath, & Borovsky, Under review). In this study, verb and noun semantic structure demonstrated systematic differences. For example, while early-learned nouns tended to demonstrate strong semantic connections with other nouns throughout vocabulary development, early-learned verbs had relatively weaker direct semantic connections to other verbs. However, this study did not consider how different semantic features may contribute to semantic structure.

In Experiment 2, we expand on the prior work showing that the semantic network structure of children’s early vocabularies demonstrates differences across feature types. Here, we measure how nouns’ and verbs’ semantic structure in children’s early vocabularies differ as a function of feature type. Importantly, we account for the differences in semantic feature composition between nouns and verbs observed in Experiment 1 and for differences in quantities of nouns and verbs in vocabularies by normalizing the semantic network measures with respect to random networks composed of each feature type with controlled numbers of nouns and verbs. In this way, we ask whether nouns and verbs differ in semantic connections to other words compared to what would be expected given the baseline feature and vocabulary composition of nouns and verbs.

### Method

#### MBCDI Vocabulary Data

Data for children’s early vocabularies came from administrations of the MBCDI vocabulary checklist (Fenson et al., 2007) stored in the WordBank MBCDI database (Frank, Braginsky, Yurovsky, & Marchman, 2017). As we were focused on understanding patterns of typical development, children were included in the data set if they had productive vocabulary size percentiles greater than the 20^th^ percentile, a commonly used cutoff separating children with typical development from those who are late talkers (e.g., Beckage et al., 2011; Feldman et al., 2005). The final sample size was 3,804 children (1437 female, 1422 male, 945 unknown).

#### Semantic Network Creation and Measurement

Semantic networks were created for each child by adding the words produced by the child as nodes. Edges between words were established if words shared semantic features. All features were included. Edges were undirected and weighted to represent the number of shared semantic features between words. For each child, separate semantic networks were created using perceptual, functional, taxonomic and encyclopedic features only. We used *graph-tool* version 2.44 (Peixoto, 2014) running on Python version 3.8.12.

**Figure 3:**
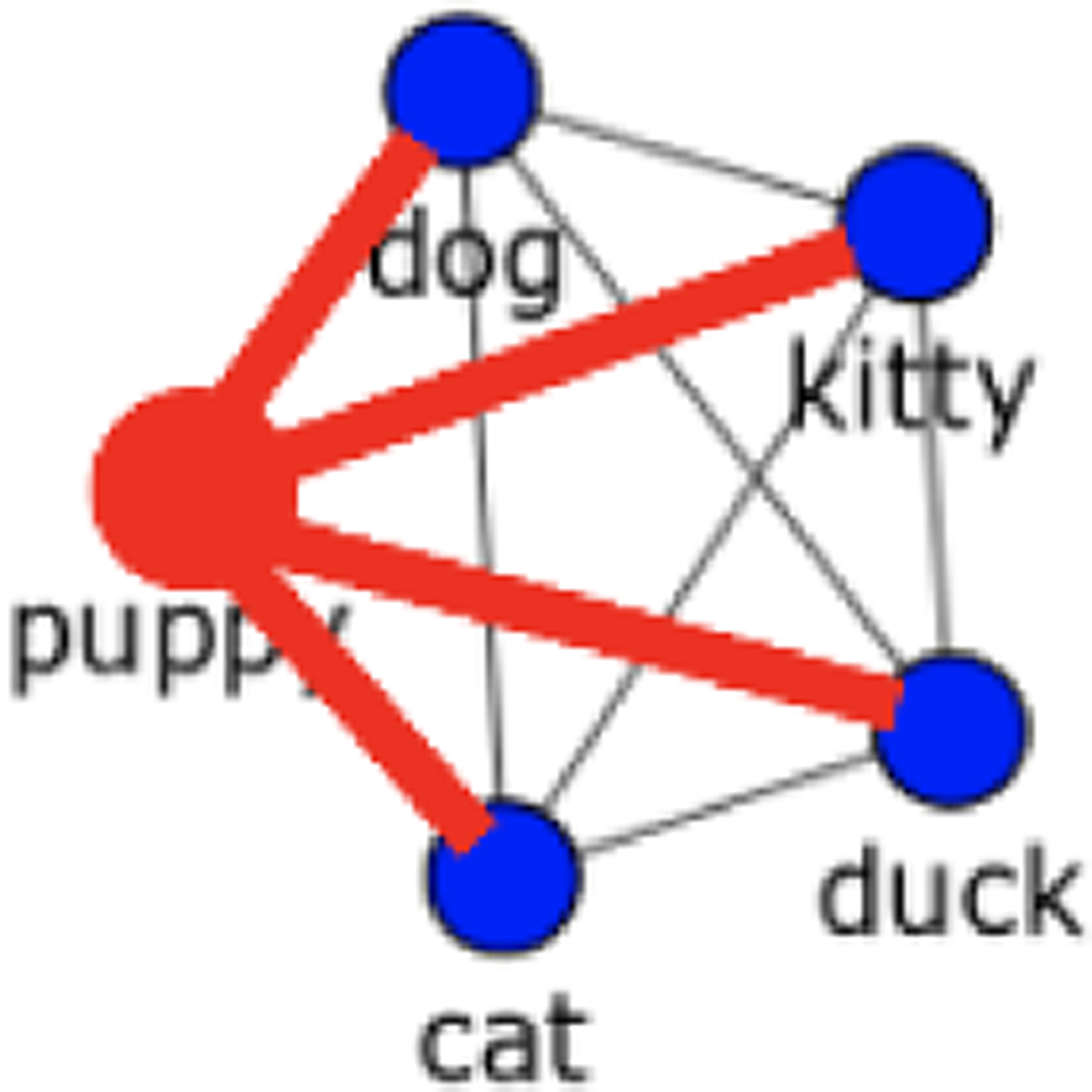
Example of weighted degree. Edges used in the calculation for “puppy” are highlighted. If all edges have a weight of 2, then the weighted degree of “puppy” is 8.

Weighted degree was measured for each word in each network for each child (see Figure 3 for an example). Weighted degree measures the sum of the weights of the edges between a node and the nodes to which it is connected. Weighted degree is high when words are strongly connected to their neighbors and low when words are unconnected or only weakly connected. Last, we calculated the average weighted degree for the nouns and for the verbs within each network type for each child.

#### Network normalization procedure

Given the result in Experiment 1 that nouns and verbs differed in the relative proportion of perceptual, functional, taxonomic, and encyclopedic features, nouns and verbs would be expected to demonstrate differences in feature-based networks. However, these differences would be relatively uninformative with respect to answering the question about whether children’s early vocabularies preferentially consist of nouns or verbs demonstrating stronger perceptual connections relative to other feature types. To address this potential limitation, we normalized the raw weighted degrees in each network type, separately for nouns and for verbs.

Another reason for normalizing the raw network measure is to account for the fact that there are different numbers of nouns and verbs on the MBCDI (or in children’s vocabularies in general, given nouns’ earlier age of acquisition). Our procedure accounts for that fact by comparing the observed network values to randomly generated networks with the same numerical composition of nouns and verbs.

Specifically, we generated 868,000 random networks across different noun-verb vocabulary sizes for each feature type. The random networks consisted of random selections of nouns and verbs from the MBCDI. Within each randomly generated network, we measured each word’s weighted degree and separately calculated the average of weighted degree for nouns and verbs across each random network. Using the distribution of weighted degree in the random networks, we calculated percentile ranks for the raw values in the children’s networks. This was done using the random network of the same noun-verb vocabulary size as the child’s network. For example, for a child with 10 nouns and three verbs, we first calculated the child’s raw weighted degree for nouns and for verbs and then compared those raw degrees to the distribution of degree for nouns and for verbs across the random networks with the same noun-verb vocabulary size.

Instead of directly sampling all random networks across the noun-verb vocabulary space (which had 3,279 distinct combinations across our sample), we strategically sampled from this space. First, we randomly sampled 15% of the noun-verb vocabulary combinations that lay within two standard deviations of the center of the space as identified with principal components analysis. Second, we sampled all noun-verb vocabulary size combinations that lay outside of the two-standard-deviations area. Third, we sampled along the edges of the noun-verb vocabulary size space. These steps resulted in a final sample of 868 points in the noun-verb vocabulary size space.

For each of the 868 noun-verb vocabulary size combinations, 1,000 randomly generated networks were created from random sets of nouns and verbs for each of the feature types. We calculated weighted degree for each noun and verb in each of these networks and then, on a network-by-network basis, calculated the average weighted degree for each part of speech. For each feature-type network, we then created a three-dimensional histogram for nouns and for verbs of the average weighted degrees across the noun-verb vocabulary space. Specifically, this was a three-dimensional array with dimensions corresponding to noun vocabulary size × verb vocabulary size × weighted degree histogram for nouns or for verbs.

The three-dimensional histogram was used to estimate unsampled points in the noun-verb vocabulary space. We linearly interpolated weighted degree across two-dimensional noun-verb vocabulary size slices of the array using the *LinearNDInterpolator* function in the Python package *scipy* version 1.8.0 (Virtanen et al., 2020). Values within the weighted degree histogram columns were then normalized so their sum was one. Last, we used kernel density estimation from the Python package *KDEpy* version 1.1.0 with bandwidth equal to two bins to slightly smooth the histograms to avoid artifacts from the binning and interpolation procedures (Odland, 2018).

The interpolated histogram was then used to normalize the raw weighted degree values from the children’s vocabulary networks. We report percentile ranks that correspond to the proportion of random networks with values at or below a child’s observed weighted degree. For example, if a child’s average weighted degree for nouns in the perceptual network was 10, we would use the interpolated histogram at the same vocabulary size for the perceptual network and calculate the proportion of randomly generated networks that had a weighted degree for nouns at or below 10. This proportion was then multiplied by 100 and served as the percentile rank that we report in our results. We call this quantity “normalized degree”.

#### Cluster analyses procedure

We used cluster-based permutation testing to compare normalized degree between nouns and verbs across vocabulary sizes in our sample (Maris & Oostenveld, 2007). This procedure identifies ranges of significantly different values in time-series-like data while controlling the family-wise error rate. Across children and for each network type, normalized degree for nouns and verbs was separately put into vocabulary size bins of 20 words. Within each network type, normalized degree for nouns and verbs was compared. Following the cluster-based permutation testing procedure, for each network-type, individual *t*-tests were conducted within each bin. Significantly different comparisons were identified using a *t* threshold of 3.29. Contiguous ranges of significantly different bins were identified and the *t* statistics of those comparisons were added together; this value is termed the cluster mass *t* statistic for that cluster. Separately, the data were randomly shuffled within children and the *t*-test procedure above was repeated 10,000 times. Last, we compared the cluster mass *t* statistic for each identified cluster in the children’s actual data to the distribution of cluster mass *t* statistics in the randomly shuffled data. This resulted in an empirical *p* value for each cluster that reflected how likely it was that the observed cluster would be as large or larger by random chance alone.

### Results

**Figure 4:**
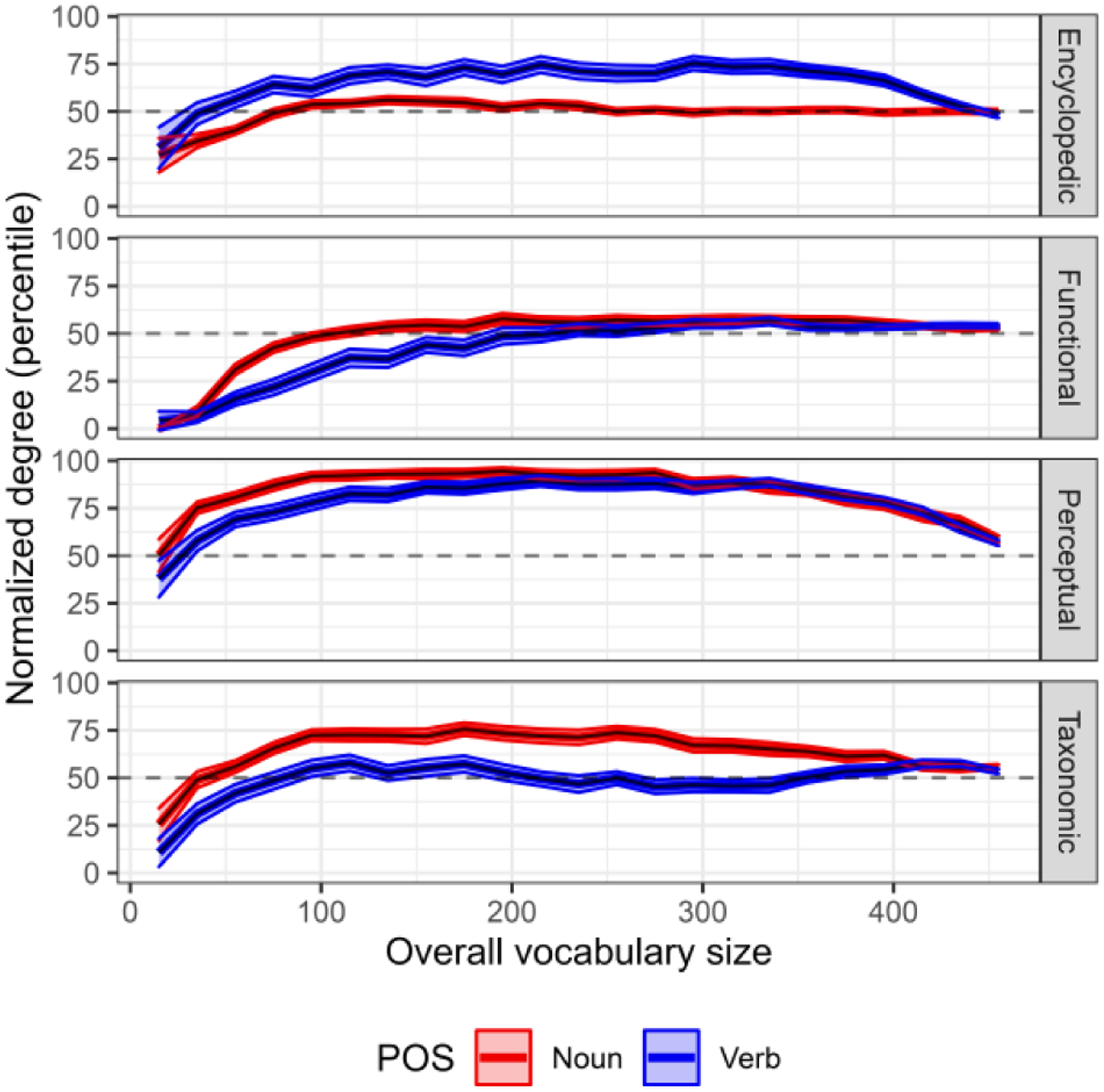
Normalized degree for nouns and verbs in semantic networks created using encyclopedic, functional, perceptual, and taxonomic features across 3,804 children’s vocabularies. Horizontal dashed line shows expected degree in randomly generated networks of the same size. Values are binned in intervals of 20 words. POS = Part of speech.

Table 3 reports the results of the cluster-based permutation testing procedure, comparing noun and verb normalized degree within each network type to identify ranges of significantly different values. Figure 4 shows the average normalized degree for nouns and verbs across children in networks created using encyclopedic, functional, perceptual, and taxonomic features. For encyclopedic features, children’s vocabularies demonstrated significantly greater normalized degree for verbs than for nouns from 20 to 420 words, nearly the entire range of vocabulary development assessed on the MBCDI. For all other features, normalized degree for nouns tended to be larger than for verbs. Specifically, for functional features, two significant clusters were identified in which noun normalized degree was higher than verb normalized degree, one from 40 to 180 words and another from 360 to 380 words. For perceptual features, a significant cluster from 20 to 180 words was identified in which normalized degree was larger for nouns than for verbs. Last, for taxonomic features, there was a significant cluster from 20 to 380 words in which normalized degree was larger for nouns than for verbs.

Last, given the enhanced role that encyclopedic features played for verbs compared to nouns and evidence for the overall importance of perceptual features for nouns, we conducted a cluster-based permutation test comparing normalized degree for encyclopedic features to normalized degree for perceptual features. For nouns, there was a significant cluster from 20 to 440 words where normalized degree for encyclopedic features was below that for perceptual features, cluster-*t* = −503.8, *p* < .001. For verbs, normalized degree for encyclopedic features was also below that for perceptual features from 80 to 440 words, cluster-*t* = −124.2, *p* < .001.

## Discussion

Verb learning is often considered to be more difficult than noun learning for young children because verbs’ referents are often less imageable and concrete than nouns’ referents (Ma et al., 2009; McDonough et al., 2011) and verbs may refer to complex transient events with many participants (Talmy, 1985). This study adds another potential reason for verbs’ difficulty – verbs’ meanings have substantial contributions from complex meanings that are not grounded in perception, object function, or taxonomic structure. Compared to nouns, verbs’ meanings are more often built from encyclopedic features, subcomponents of meaning often related to social relationships, affect/emotion, and decontextualized knowledge (Cree & McRae, 2003). Moreover, despite the difficulty of verbs’ meanings, children’s early verb vocabularies demonstrated semantic relationships with other words through encyclopedic features in addition to perceptual features. Rather than avoiding the challenge of verbs’ complex meanings, children systematically used these complex features to structure their vocabularies.

At the same time, perceptual information also supported structure of both nouns and verbs. This finding provides some support for theories that prioritize the impact of perceptual information in early lexical representation, which have focused largely on evidence in nouns. For example, Quinn and Eimas (1997) argued that meaning representations are initially grounded in perceptual information, which later form the basis for more advanced conceptual representations. The findings are also consistent with embodied cognition accounts, which posit that representations of word meaning are grounded in sensorimotor representations (Wellsby & Pexman, 2014).

While perceptual information supported structure of both verb and noun networks, the structure of verb semantic networks was additionally driven by complex encyclopedic information. This pattern suggests that conceptual development may proceed differently in response to learning nouns compared to verbs. Given that verbs refer to entities that are not pre-individuated in the world (Gentner, 1982), fundamental differences between nouns and verbs may produce different perceptual-conceptual demands. Alternatively, it is possible that the shift from perceptual to conceptual information had already occurred in our sample; by the time these children had learned their first verbs, they had already learned many nouns.

These findings offer a rich starting point for future work. For example, future studies could determine whether there are subtypes of encyclopedic or perceptual features that exert disproportionately strong effects on children’s vocabulary structure. Additionally, future work should directly address how the semantic-feature differences seen here affect noun and verb learning by, for instance, examining how age of acquisition of nouns and verbs differs as a function of these words’ investment in perceptual vs. encyclopedic information.

In sum, this study offers a first look at the contribution of semantic feature type to children’s early noun and verb semantics. While noun meanings tended to prioritize directly perceptible information, verb meanings reflected more complex encyclopedic information even from the very beginnings of vocabulary development.

## Figures and Tables

**Table 1:** Experiment 1 model results.

	df	SS	MS	F	*p*
FT	3	20.9	7.0	261.5	<.001 ***
POS	1	0.0	0.0	0.0	1
FT × POS	3	10.1	3.4	125.7	<.001 ***
Residuals	1840	49.1	0.0		

*Note*. FT = Feature type; POS = Part of speech.

*** *p* < .001

**Table 2:** Mean proportions by feature type.

	Mean (SE)	
Feature type	Noun	Verb
Encyclopedic	0.27 (0.01)	0.55 (0.02)
Functional	0.20 (0.01)	0.03 (0.02)
Perceptual	0.41 (0.01)	0.28 (0.02)
Taxonomic	0.12 (0.01)	0.14 (0.02)

**Table 3:** Experiment 2 results

Type	Range	Direction	Cluster *t*
Encyclopedic	20–420	V > N	−181.5
Functional	40–180	N > V	55.2
	360–380	N > V	7.6
Perceptual	20–180	N > V	46.6
Taxonomic	20–380	N > V	161.0

*Note*. N = Noun; V = Verb. Range is number of words. All cluster comparisons have *p* values < .001.

## References

[R1] Alishahi A, & Stevenson S (2010). A computational model of learning semantic roles from child-directed language. Language and Cognitive Processes, 25(1), 50–93. doi: 10.1080/01690960902840279

[R2] Beckage NM, & Colunga E (2019). Network growth modeling to capture individual lexical learning. Complexity, 2019, 1–17. doi: 10.1155/2019/7690869

[R3] Beckage NM, Smith L, & Hills T (2011). Small worlds and semantic network growth in typical and late talkers. PLoS ONE, 6(5). doi: 10.1371/journal.pone.0019348 PMC3092758 21589924

[R4] Borovsky A (2020). When slowing down processing helps learning: Lexico‐semantic structure supports retention, but interferes with disambiguation of novel object‐label mappings. Developmental Science. doi: 10.1111/desc.12963 PMC8128105 32160363

[R5] Borovsky A (2022). Lexico-semantic structure in vocabulary and its links to lexical processing in toddlerhood and language outcomes at age three. Developmental Psychology, 58(4), 607–630. (2022-47461-001). doi: 10.1037/dev0001291 35343711 PMC9734010

[R6] Borovsky A, Ellis EM, Evans JL, & Elman JL (2016). Lexical leverage: Category knowledge boosts real-time novel word recognition in 2-year-olds. Developmental Science, 19(6), 918–932. doi: 10.1111/desc.12343 26452444 PMC4826629

[R7] Borovsky A, Peters RE, Cox J, & McRae K (Under review). Feats: A database of semantic features for early produced noun concepts. 10.3758/s13428-023-02242-x PMC11630474 38148439

[R8] Childers JB, Parrish R, Olson CV, Burch C, Fung G, & McIntyre KP (2016). Early verb learning: How do children learn how to compare events? Journal of Cognition and Development, 17(1), 41–66. doi: 10.1080/15248372.2015.1042580 27092030 PMC4833120

[R9] Cree GS, & McRae K (2003). Analyzing the factors underlying the structure and computation of the meaning of chipmunk, cherry, chisel, cheese, and cello (and many other such concrete nouns). Journal of Experimental Psychology: General, 132(2), 163–201. doi: 10.1037/0096-3445.132.2.163 12825636

[R10] Della Rosa PA, Catricalà E, Vigliocco G, & Cappa SF (2010). Beyond the abstract—concrete dichotomy: Mode of acquisition, concreteness, imageability, familiarity, age of acquisition, context availability, and abstractness norms for a set of 417 Italian words. Behavior Research Methods, 42(4), 1042–1048. doi: 10.3758/BRM.42.4.1042 21139171

[R11] Dowty D (1991). Thematic proto-roles and argument selection. Language, 67(3), 547–619.

[R12] Engelthaler T, & Hills TT (2017). Feature biases in early word learning: Network distinctiveness predicts age of acquisition. Cognitive Science, 41, 120–140. doi: 10.1111/cogs.12350 26923664

[R13] Feldman HM, Dale PS, Campbell TF, Colborn DK, Kurs-Lasky M, Rockette HE, & Paradise JL (2005). Concurrent and predictive validity of parent reports of child language at ages 2 and 3 years. Child Development, 76(4), 856–868. doi: 10.1111/j.1467-8624.2005.00882.x 16026501 PMC1350485

[R14] Fenson L, Marchman VA, Thal D, Dale P, Reznick JS, & Bates E (2007). MacArthur-Bates Communicative Development Inventories: User’s guide and technical manual (2nd Edition). Baltimore, MD: Brookes Publishing Co.

[R15] Fourtassi A, Bian Y, & Frank MC (2020). The growth of children’s semantic and phonological networks: Insight from 10 languages. Cognitive Science, 44(7). doi: 10.1111/cogs.12847 32621305

[R16] Frank MC, Braginsky M, Yurovsky D, & Marchman VA (2017). Wordbank: An open repository for developmental vocabulary data. Journal of Child Language, 44(3), 677–694. doi: 10.1017/S0305000916000209 27189114

[R17] Frank MC, Braginsky M, Yurovsky D, & Marchman VA (2021). Variability and consistency in early language learning: The Wordbank project. Cambridge, MA: MIT Press. Retrieved from https://langcog.github.io/wordbank-book/index.html

[R18] Gentner D (1982). Why nouns are learned before verbs: Linguistic relativity versus natural partitioning. In Kuczaj SA (Ed.), Language development: Vol. 2. Language, thought and culture (pp. 301–334). Hillsdale, NJ: Erlbaum.

[R19] Gilhooly KJ, & Logie RH (1980). Age-of-acquisition, imagery, concreteness, familiarity, and ambiguity measures for 1,944 words. Behavior Research Methods & Instrumentation, 12(4), 395–427. doi: 10.3758/BF03201693

[R20] Hansen P (2017). What makes a word easy to acquire? The effects of word class, frequency, imageability and phonological neighbourhood density on lexical development. First Language, 37(2), 205–225. doi: 10.1177/0142723716679956

[R21] Haryu E, Imai M, & Okada H (2011). Object similarity bootstraps young children to action-based verb extension. Child Development, 82(2), 674–686. doi: 10.1111/j.1467-8624.2010.01567.x 21410924

[R22] Hills TT, Maouene J, Riordan B, & Smith LB (2010). The associative structure of language: Contextual diversity in early word learning. Journal of Memory and Language, 63(3), 259–273. doi: 10.1016/j.jml.2010.06.002 20835374 PMC2936494

[R23] Hills TT, Maouene M, Maouene J, Sheya A, & Smith L (2009). Longitudinal analysis of early semantic networks: Preferential attachment or preferential acquisition? Psychological Science, 20(6), 729–739. doi: 10.1111/j.1467-9280.2009.02365.x 19470123 PMC4216730

[R24] Inhelder B, & Piaget J (1964). The growth of logic in the child. London, UK: Routledge & Kegan Paul.

[R25] Kersten AW, & Smith LB (2002). Attention to novel objects during verb learning. Child Development, 73(1), 93–109. doi: 10.1111/1467-8624.00394 14717246

[R26] Kueser JB, Horvath S, & Borovsky A (In prep.). Semantic feature norms for early-learned English verbs.

[R27] Kueser JB, Horvath S, & Borovsky A (Under review). Two pathways in vocabulary development: Large-scale differences in noun and verb semantic structure. 10.1016/j.cogpsych.2023.101574 PMC10832511 37209501

[R28] Lenth R (2019). emmeans: Estimated marginal means, aka least-squares means. Retrieved from https://CRAN.R-project.org/package=emmeans

[R29] Ma W, Golinkoff RM, Hirsh-Pasek K, Mcdonough C, & Tardif T (2009). Imageability predicts the age of acquisition of verbs in Chinese children. Journal of Child Language, 36(2), 405–423. doi: 10.1017/S0305000908009008 18937878 PMC2925137

[R30] Maris E, & Oostenveld R (2007). Nonparametric statistical testing of EEG- and MEG-data. Journal of Neuroscience Methods, 164(1), 177–190. doi: 10.1016/j.jneumeth.2007.03.024 17517438

[R31] McDonough C, Song L, Hirsh-Pasek K, Golinkoff RM, & Lannon R (2011). An image is worth a thousand words: Why nouns tend to dominate verbs in early word learning. Developmental Science, 14(2), 181–189. doi: 10.1111/j.1467-7687.2010.00968.x 21359165 PMC3043374

[R32] McRae K, Cree GS, Seidenberg MS, & McNorgan C (2005). Semantic feature production norms for a large set of living and nonliving things. Behavior Research Methods, 37(4), 547–559. doi: 10.3758/BF03192726 16629288

[R33] McRae K, Ferretti T, & Amyote L (1997). Thematic roles as verb-specific concepts. Language and Cognitive Processes, 12(2), 137–176. doi: 10.1080/016909697386835

[R34] Meints K, Plunkett K, & Harris PL (2008). Eating apples and houseplants: Typicality constraints on thematic roles in early verb learning. Language and Cognitive Processes, 23(3), 434–463. doi: 10.1080/01690960701726232

[R35] Odland T (2018). tommyod/KDEpy: Kernel density estimation in Python. Zenodo. doi: 10.5281/zenodo.2392268

[R36] Peixoto TP (2014). The graph-tool python library. Figshare. doi: 10.6084/m9.figshare.1164194

[R37] Peters RE, & Borovsky A (2019). Modeling early lexicosemantic network development: Perceptual features matter most. Journal of Experimental Psychology: General, 148(4), 763–782. doi: 10.1037/xge0000596 30973265 PMC6461380

[R38] Peters RE, Kueser JB, & Borovsky A (2021). Perceptual connectivity influences toddlers’ attention to known objects and subsequent label processing. Brain Sciences, 11(2), 163. doi: 10.3390/brainsci11020163 33513707 PMC7912090

[R39] Pexman PM, Hargreaves IS, Siakaluk PD, Bodner GE, & Pope J (2008). There are many ways to be rich: Effects of three measures of semantic richness on visual word recognition. Psychonomic Bulletin & Review, 15(1), 161–167. doi: 10.3758/PBR.15.1.161 18605497

[R40] Ponari M, Norbury CF, & Vigliocco G (2018). Acquisition of abstract concepts is influenced by emotional valence. Developmental Science, 21(2), e12549. doi: 10.1111/desc.12549 28224689

[R41] Ponari M, Norbury CF, & Vigliocco G (2020). The role of emotional valence in learning novel abstract concepts. Developmental Psychology, 56(10), 1855–1865. doi: 10.1037/dev0001091 32700948

[R42] Pruden SM, Hirsh-Pasek K, Golinkoff RM, & Hennon EA (2006). The birth of words: Ten-month-olds learn words through perceptual salience. Child Development, 77(2), 266–280. doi: 10.1111/j.1467-8624.2006.00869.x 16611171 PMC4621011

[R43] Quinn PC, & Eimas PD (1997). A reexamination of the perceptual-to-conceptual shift in mental representations. Review of General Psychology, 1(3), 271–287.

[R44] Quinn PC, & Eimas PD (2000). The emergence of category representations during infancy: Are separate perceptual and conceptual processes required? Journal of Cognition and Development, 1(1), 55–61. doi: 10.1207/S15327647JCD0101N_6

[R45] R Development Core Team. (2008). R: A language and environment for statistical computing. Vienna, Austria: R Foundation for Statistical Computing. Retrieved from http://www.R-project.org

[R46] Sailor KM (2013). Is vocabulary growth influenced by the relations among words in a language learner’s vocabulary? Journal of Experimental Psychology: Learning, Memory, and Cognition, 39(5), 1657–1662. doi: 10.1037/a0032993 23647381

[R47] Smiley SS, & Brown AL (1979). Conceptual preference for thematic or taxonomic relations: A nonmonotonic age trend from preschool to old age. Journal of Experimental Child Psychology, 28(2), 249–257. doi: 10.1016/0022-0965(79)90087-0

[R48] Smolík F (2019). Imageability and neighborhood density facilitate the age of word acquisition in Czech. Journal of Speech, Language, and Hearing Research, 62(5), 1403–1415. doi: 10.1044/2018_JSLHR-L-18-0242 31046539

[R49] Stella M, Beckage NM, Brede M, & De Domenico M (2018). Multiplex model of mental lexicon reveals explosive learning in humans. Scientific Reports, 8(1), 2259. doi: 10.1038/s41598-018-20730-5 29396497 PMC5797130

[R50] Steyvers M, & Tenenbaum JB (2005). The large-scale structure of semantic networks: Statistical analyses and a model of semantic growth. Cognitive Science, 29(1), 41–78. doi: 10.1207/s15516709cog2901_3 21702767

[R51] Talmy L (1985). Lexicalization patterns: Semantic structure in lexical forms. In Shopen T (Ed.), Language typology and syntactic description, Vol. III: Grammatical categories and the lexicon (pp. 57–149). Cambridge: Cambridge University Press.

[R52] Vinson DP, & Vigliocco G (2008). Semantic feature production norms for a large set of objects and events. Behavior Research Methods, 40(1), 183–190. doi: 10.3758/BRM.40.1.183 18411541

[R53] Virtanen P, Gommers R, Oliphant TE, Haberland M, Reddy T, Cournapeau D, … SciPy 1.0 Contributors. (2020). SciPy 1.0: Fundamental Algorithms for Scientific Computing in Python. Nature Methods, 17, 261–272. doi: 10.1038/s41592-019-0686-2 32015543 PMC7056644

[R54] Wellsby M, & Pexman PM (2014). Developing embodied cognition: Insights from children’s concepts and language processing. Frontiers in Psychology, 5. doi: 10.3389/fpsyg.2014.00506 PMC4036138 24904513

[R55] Yuan S, Fisher C, Kandhadai P, & Fernald A (2011). You can stipe the pig and nerk the fork: Learning to use verbs to predict nouns. In Proceedings of the 35th annual Boston University conference on language development (pp. 665–677).

[R56] Zelazo PR, & Kearsley RB (1980). The emergence of functional play in infants: Evidence for a major cognitive transition. Journal of Applied Developmental Psychology, 1(2), 95–117. doi: 10.1016/0193-3973(80)90002-7

